# Assessment of artificial intelligence (AI) reporting methodology in glioma MRI studies using the Checklist for AI in Medical Imaging (CLAIM)

**DOI:** 10.1007/s00234-023-03126-9

**Published:** 2023-02-07

**Authors:** Abhishta Bhandari, Luke Scott, Manuela Weilbach, Ravi Marwah, Arian Lasocki

**Affiliations:** 1 Townsville University Hospital, 100 Angus Smith Drive, Townsville, QLD 4814 Australia; 2 School of Medicine and Dentistry, James Cook University, 1 James Cook Drive, Townsville, QLD 4814 Australia; 3 Cairns Hospital, 165 Esplanade, Cairns, QLD 4870 Australia; 4 Redcliffe Hospital, Anzac Avenue, Redcliffe, QLD 4020 Australia; 5 Department of Cancer Imaging, Peter MacCallum Cancer Centre, Melbourne, Victoria Australia; 6 Sir Peter MacCallum Department of Oncology, The University of Melbourne, Melbourne, Victoria Australia

**Keywords:** Artificial intelligence, Machine learning, Deep learning, Quality, Glioma

## Abstract

**Purpose:**

The Checklist for Artificial Intelligence in Medical Imaging (CLAIM) is a recently released guideline designed for the optimal reporting methodology of artificial intelligence (AI) studies. Gliomas are the most common form of primary malignant brain tumour and numerous outcomes derived from AI algorithms such as grading, survival, treatment-related effects and molecular status have been reported. The aim of the study is to evaluate the AI reporting methodology for outcomes relating to gliomas in magnetic resonance imaging (MRI) using the CLAIM criteria.

**Methods:**

A literature search was performed on three databases pertaining to AI augmentation of glioma MRI, published between the start of 2018 and the end of 2021

**Results:**

A total of 4308 articles were identified and 138 articles remained after screening. These articles were categorised into four main AI tasks: grading (*n*= 44), predicting molecular status (*n*= 50), predicting survival (*n*= 25) and distinguishing true tumour progression from treatment-related effects (*n*= 10). The average CLAIM score was 20/42 (range: 10–31). Studies most consistently reported the scientific background and clinical role of their AI approach. Areas of improvement were identified in the reporting of data collection, data management, ground truth and validation of AI performance.

**Conclusion:**

AI may be a means of producing high-accuracy results for certain tasks in glioma MRI; however, there remain issues with reporting quality. AI reporting guidelines may aid in a more reproducible and standardised approach to reporting and will aid in clinical integration.

**Supplementary Information:**

The online version contains supplementary material available at 10.1007/s00234-023-03126-9.

## Introduction

Gliomas are the most common primary malignant intracranial tumours and are associated with a poor prognosis. Imaging plays a key role in the diagnosis and management of patients with gliomas. Artificial intelligence (AI) methodologies have been used as a tool to extract quantitative data from imaging modalities, in particular magnetic resonance imaging (MRI). Aspects of glioma diagnosis and management that have been previously examined include prediction of pseudoprogression [1], grade [2], molecular status [3] and survival [4]. Studies have demonstrated high performance, sensitivities and specificities for these tasks and thus, clinical integration of AI may be of use. Information gained through these algorithms may aid clinicians in counselling on prognosis, guide preoperative management, overcome the limitations of histology and guide post-treatment follow-up [5].

For optimal clinical translatability, there remain issues with the development and reporting of AI algorithm methods [6]. Reproducibility of results has been a challenge; hence, rigour of experimental design and reporting is of importance to ensure generalisability for clinical practice [7]. Guidelines have been developed to improve the quality of AI algorithm reporting within the literature. Such guidelines include the CONSORT-AI (Reporting Guidelines for Clinical Trial Protocols for Interventions Involving Artificial Intelligence) [8], SPIRIT-AI (Reporting Guidelines for Clinical Trial Reports for Interventions Involving Artificial Intelligence) [9] and the recently announced QUADAS-AI (Quality Assessment of Diagnostic Accuracy Studies Artificial Intelligence) [10]. In particular, the Checklist for AI in Medical Imaging (CLAIM) [11] is a 42-item checklist comprising elements to evaluate optimal reproducibility, rigour, quality and generalisability. This is viewed as a “best practice” guideline for reporting AI algorithms within the literature. The application of this checklist to the current literature may provide insights into the progress and reporting quality of AI algorithms in glioma imaging, in turn aiding clinical integration. The aim of this study is to perform a thorough methodological examination of the recently published literature for AI tasks involving glioma MRI as an example of using the CLAIM criteria [11].

## Methods

### Search strategy

The literature search followed the Preferred Reporting Items for Systematic Reviews and Meta-Analyses (PRISMA) guidelines and was performed on 04/01/2022. In order to focus on the most recent literature, we only examined studies from the start of 2018 to the end of 2021. This was also chosen as we wanted to examine studies 2 years before the CLAIM introduction in 2020, and 2 years after its introduction. Search terms were derived by pilot searches of the literature, the PICO (population, intervention, comparison, outcomes) framework and search of Medical Subject Headings (MeSH) subheadings by the first author (A.P.B.). The search was performed on three databases: PubMed, Scopus and Web of Science. An additional literature search following PRISMA guidelines was also performed independently by the third author (M.W.) on 01/02/2022 in consultation with a hospital librarian. Terms are detailed as follows, with adaptations made for each database: (*“machine learning” OR “artificial intelligence” OR “support vector machine” OR “convolutional neural network” OR “deep learning”) AND (brain OR cerebrum) AND (MRI OR “magnetic resonance”) AND (tumour OR tumor OR cancer OR carcinoma OR neoplasm OR glioma*).

### Selection of studies

Original studies reporting AI outcomes derived from glioma MRI in adult patients were included. Outcomes included grading, response to treatment, survival and molecular status. Studies were excluded if they were non-English language, case reports, literature reviews, conference abstracts, preliminary studies, lecture notes or paediatric studies. In addition, technique-based studies (such as segmentation or studies focusing on pre-processing methodology), comparative studies and studies on MRI-based identification of gliomas were excluded.

### Outcomes and data extraction

The first, second and third author (A.P.B., L.S. and M.W.) split and independently assessed the included articles according to the 42-item CLAIM checklist [11]. Subsequently, the first, second and third authors cross-checked each other’s articles and conflicts were resolved by group discussion. Additional data recorded included the type of journal (computer science, medical or non-medical science), the derived aim of the study, the AI algorithm and the results of the highest performing pipeline. The validation set was reported first; if this was not available, the testing set was reported.

### Data analysis

Descriptive statistics were generated using the MedCalc 19.4.1 statistical software.

## Results

A total of 4308 articles were obtained, comprising 789 articles from PubMed, 2141 articles from Scopus (conference abstracts were excluded) and 1378 articles from Web of Science. After duplicates were removed, 2178 articles remained. Articles were then screened by abstract and title, and following this, 384 articles remained. After full texts were reviewed, 119 articles were considered to fit the inclusion/exclusion criteria. The additional literature search by the third author (M.W.) identified a further 19 articles. This resulted in a total of 138 articles. Of these, 25 articles were published in 2018, 36 in 2019, 38 in 2020 and 39 in 2021.

The majority of articles investigated one of four AI tasks: 50 articles evaluated algorithms predicting molecular status (such as isocitrate dehydrogenase (IDH) or 1p/19q status), 44 articles examined grading, 25 articles assessed survival and 10 articles examined true tumour progression (TTP) versus treatment-related effects (TRE). Nine articles assessed other tasks not included under the previously specified groups. The Cancer Imaging Archive (TCIA) was utilised by 31 articles and the Brain Tumor Segmentation (BraTS) challenge by 30 articles.

For predicting tumour grade, reported AUCs (areas under the curve) ranged from 0.72 to 1. The highest performance for grading was obtained by De Looze et al., who differentiated between WHO CNS grade 2 and 4 gliomas (AUC=1, sensitivity=100%, specificity=100%) [12]. However, differentiating grade 2 and 3 gliomas yielded AUC of 0.88, sensitivity 82% and specificity 94%, while for distinguishing grade 3 and 4 gliomas, AUC, sensitivity and specificity were 0.97, 100% and 97%, respectively. For molecular status, reported AUCs ranged from 0.70 to 0.99. Yogananda et al. obtained the highest AUC=0.99 (sensitivity=98%, specificity=97%) for IDH prediction [13]. AUCs ranged from 0.58 to 0.98 for survival prediction. Su et al. achieved the best performance, predicting glioblastoma (GBM) survival beyond a 6-month period with an AUC=0.98 (sensitivity=93.3% and specificity=96.7%) [14]. For TRE versus TTP, reported AUCs ranged from 0.8 to 0.94, with the highest result obtained by Elshafeey et al. (AUC=0.94) [15].

**Fig. 1:**
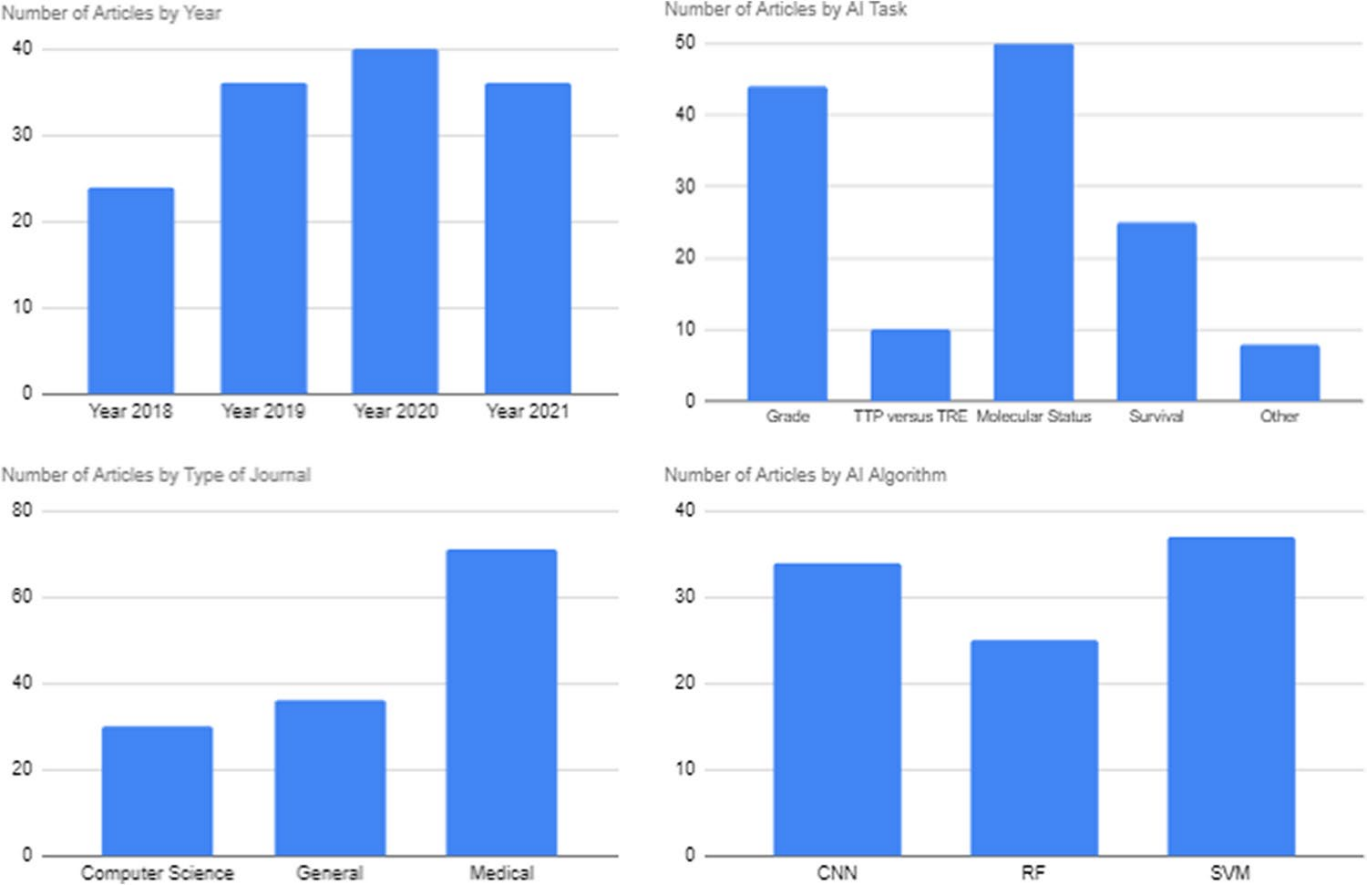
Number of articles by year, type of journal, AI task and 3 most frequently utilised algorithms (abbreviations - TTP true tumour progression, TRE treatment-related effects, CNN convolutional neural network, RF random forest, SVM support vector machine)

Most articles were published in medical journals (71 articles), followed by non-medical science journals (37 articles) and computer science journals (30 articles). The 3 most frequently utilised AI algorithms were convolutional neural networks (CNN; utilised in 34 articles), random forest (RF; used in 26 articles) and supported vector machine (SVM; utilised in 37 articles) Fig 1.

**Fig. 2:**
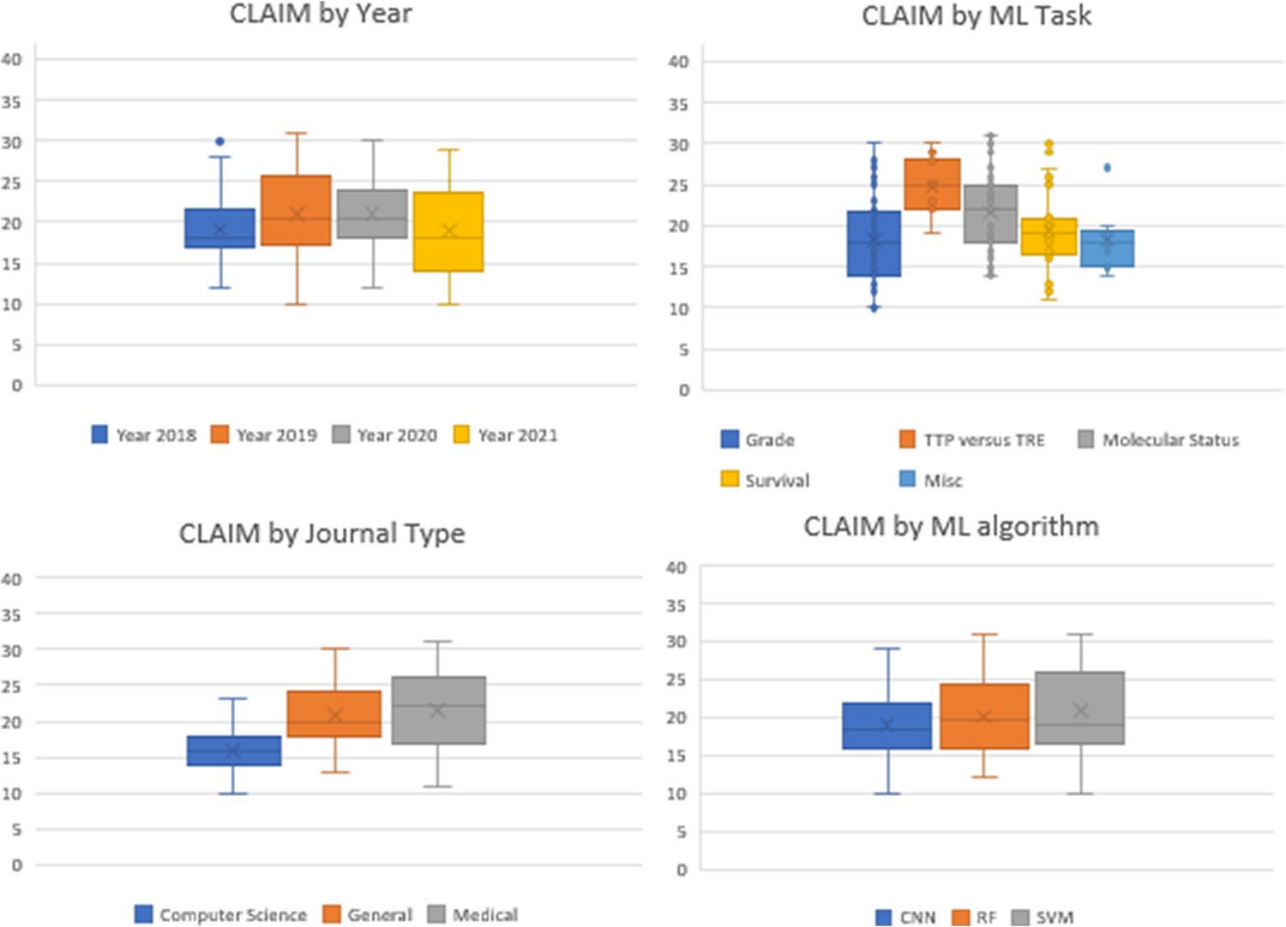
CLAIM scores by year, AI task, journal type and 3 most frequently utilised AI algorithms (abbreviations - TTP true tumour progression, TRE treatment-related effects, CNN convolutional neural network, RF random forest, SVM support vector machine)

The average CLAIM score was 20 out of 42 (48%, range: 10–31). Examining CLAIM compliance by year, the average score for 2018 was 19/42 (45%, range: 12–30), for 2019 was 21/42 (50%, range: 10–31), for 2020 was 21/42 (50%, range: 12–30) and for 2021 was 18/42 (43%, range: 10–29). There was no significant difference between CLAIM compliance based on year (ANOVA *p*-value=0.2). For AI tasks, the highest CLAIM compliance was seen in TTP versus TRE - average: 24/42 (57%, range: 19–30), followed by molecular status - average: 21/42 (50%, range: 14–31), survival - average: 19/42 (45%, range: 11–30), grade - average: 18/42 (43%, range: 10–30) and those that did not fit within one of the above categories (average: 18/42: 43%, range: 14–27). Medical journals had the highest CLAIM compliance (average 21/42: 50%, range: 11–31), followed by non-medical science journals (average: 20/42, 48%, range: 13–30) and computer science (average: 16/42, 38%, range: 10–23). For the 3 most frequently utilised algorithms, the average CLAIM compliance for CNNs was 18/42 (range 10–29), RF was 20/42 (48%, range: 12–31) and SVM was 20/42 (48%, range 10–31). Figure 2 demonstrates these findings graphically in box and whisker plots.

**Fig. 3:**
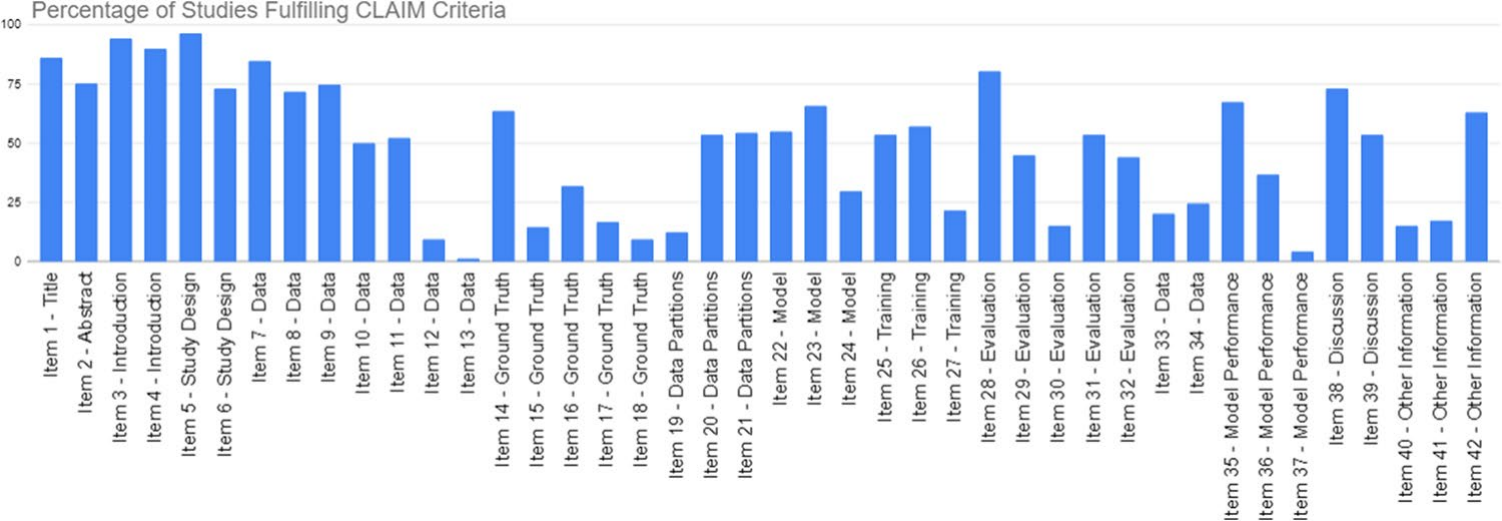
Percentage of studies fulfilling CLAIM criteria by item number

Figure 3 demonstrates the percentage of studies fulfilling each CLAIM criterion. The highest CLAIM compliance percentages were seen in the initial criteria such as the title/abstract, introduction and reporting of study design. The highest compliance item was seen in item 3, which assesses the reporting of scientific and clinical background, and the clinical role of the AI approach. Common areas for improvement identified include the reporting of data sources, ground truth and validation. The poorest performing subsection was the reporting of ground truth. For example few studies explicitly stated that neuropathologists were involved in the histological diagnosis or utilised a scoring system for inter-observer variability between neuropathologists. Of the 138 articles, only 50% of articles were externally validated. The lowest CLAIM compliance was observed for item 13, which assesses the reporting of missing data. Compared to computer science journals, medical journals were more likely to discuss clinical implications for practice. Medical journals were also more likely to have a more structured abstract than non-medical science journals.

We found no association between CLAIM score and citation count (Pearson’s correlation coefficient=0.01). There was no association between CLAIM score and performance of the algorithm (Pearson’s correlation coefficient=0). There were no significant differences in CLAIM scores between AI tasks (ANOVA *p*-value=0.029, where Bonferroni corrected *p*>0.01 means no significance).

## Discussion

AI algorithms in the current literature are achieving a high level of performance in terms of AUC (either under a receiver operator or precision-recall curve), sensitivity, specificity and accuracy. However, with an average CLAIM score of 20/42 (48%; range 10–31), there have been areas identified for improvement which affect reproducibility and generalisability. There were minimal differences in CLAIM score seen by year, AI task, journal type and top three AI algorithms (by number). Of particular note, quality is not improving over time. Pre-CLAIM (years 2018 and 2019) and post-CLAIM (years 2020 and 2021) showed no change in CLAIM adherence. The primary areas of improvement in CLAIM scoring were in reporting of the ground truth and data use (such as reporting how missing data was handled and how data was de-identified). There was no significant difference in scores between articles published before or after the introduction of CLAIM in the aforementioned areas. This may be due to the recency of the CLAIM criteria such that publishers may be unaware of its existence. Of note, no included studies reported quality utilising CLAIM. Further studies should consider reporting quality with the criteria to allow for a standardised and reproducible approach from which comparisons may be drawn. In addition, there were noteworthy differences in the structure of the included articles, and emphasis was placed on different areas depending on whether the study was reported in a computer science versus medical journal. For example, the greatest difference between these two journal types was that medical journals placed more emphasis on clinical implications.

Using the CLAIM criteria against these studies has highlighted that many pieces of information needed to reach clinical practice are not reported. There are, however, items of the checklist that are not applicable to certain papers. The major areas of deficiency in the data section were the de-identification process (item 12) and handling of missing data (item 13), where only about 8% and 1% of papers reported these respectively. The primary implication of this is the ethical consequences if there were any data leaks and thereby releasing patient information [16, 17]. Both can also affect the statistical analysis of the AI algorithm and thus cause misinterpretation of model performance [17]. However, as there were many studies that utilised open-access databases such as the TCIA, the process of de-identification (and even handling of missing data to an extent) is not applicable.

To accurately predict model performance, a well-defined ground truth is needed, including exact definitions from radiologists, a rationale for why these measurements are the standard for a supervised model to learn from, and methods for dispute resolution ensure a robust gold standard [18]. However, nearly all items in the ground truth section had a compliance of below 50%. Approximately 17% included a rationale for choice of ground truth (item 15), and 11% included methods to measure inter- and intra-rater variability and steps to reduce or mitigate them. This ultimately affects the interpretability and overall accuracy of the model, and should be discussed as a limitation by authors.

The model section achieved adequate reporting standards for the most part. Although 28% of authors indicated information regarding how their models were initialised (item 24), this is only applicable to deep learning AI programs [17]. This item is not applicable to many of the articles, as most used traditional machine learning models. After adjusting for this, the compliance would be high.

Almost half the studies (53%) assessed did not include the necessary information to duplicate their models (item 25). Without this, many of these articles cannot undergo rigorous testing necessary to be implemented into clinical practice [19]. In training a model, however, there are many instances where there is only one model used and therefore ensembling is not utilised. While approximately 24% of studies included a description of ensemble methods, this would not have been applicable to many others.

For stakeholders to be confident in the performance of the algorithm, evaluation of the model needs to be of a high standard. This being the validation of the model against an external source, or if it did not, explaining this as a limitation (item 32), of which only approximately 53% did so. Only about 17% of authors performed a robustness/sensitivity analysis of their models (item 30), meaning that the validity of their performance is often unclear [19].

A limitation of AI models and algorithms is that it needs an adequate sample of data, and can only interpret what it has been taught. This also includes demographic information of patients. If this is not known, the program cannot confidently predict outcomes of, for example, molecular status or survival in gliomas. With only approximately 31% of authors including this information (item 34), it can make it difficult to reproduce the model performance and compare to different populations [17]. Limitation in providing this information, however, is that patient information is not being readily available in open-access databases such as the TCIA.

For the clinical integration of AI, authors and stakeholders must understand where and why a model fails, producing false negatives and false positives. With only approximately 3% of authors accounting for failures through a confusion matrix, a very small portion of papers help to better understand the strengths and, more importantly, limitations and areas for improvement for algorithms [19]. Another area for improvement is the accessibility to the model’s full protocol (item 41), where only 25% of authors included a link. This item may help researchers access and further improve the algorithm by adding other demographic information that may broaden the algorithm’s applicability to multiple centres or areas. Websites such as GitHub are also known to facilitate the uptake and sharing of code.

Three other studies have evaluated the use of CLAIM to assess AI reporting quality in other fields and observed very similar results to this article. O’Shea et al. evaluated 186 articles using CNNs for cancer in general, [7] while Le VNT et al. assessed 6 articles using CNNs for the detection of odontogenic cysts [20]. Lastly, Belue et al. evaluated 53 articles on the detection and classification of prostate cancers utilising AI MRI imaging applications [21]. Belue et al. identified reporting items which were not applicable to certain studies and accounted for this in their analysis, but nevertheless identified similar issues and areas for improvement as our article and the other two aforementioned articles. Major opportunities identified for improvement [7, 20, 21] included the handling of data, and reporting of ground truth across all areas, including a well-defined definition and evaluation of models. We have expanded on the current literature by evaluating models other than CNNs, in tasks that are most progressed within the literature, and in a large sample of articles that are specific to gliomas. Ongoing appraisal of the quality and areas of improvement of the existing literature is a necessary step in the process of translating AI research into clinical practice. Indeed, this is reflected in the “Position Statement on the Regulation of AI in Medicine” by the Royal Australian and New Zealand College of Radiologists (RANZCR) [22]. Recommendation two of three states that AI systems must be proven to an appropriate standard of evidence and deemed safe in the clinical context in which they are intended to be applied. Using guidelines such as CLAIM will help ensure such recommendations are met. In addition, the Food and Drug Administration (FDA) Centre for Devices and Radiological Health has also released a regulatory framework that would allow for use in real world environments while ensuring efficacy and safety [23].

Our study has some limitations. Firstly, the CLAIM criteria were only developed in 2020, and thus, the authors of many included studies did not have the opportunity to incorporate the criteria into their work at commencement. Importantly, however, our study demonstrates that authors did not adopt reporting items prior to the introduction of the CLAIM criteria and are still yet to adopt the guideline. As the criteria gain popularity and become a point of reference for authors, we hope to see greater adherence and improved research quality. Of note, our study only includes papers assessing gliomas, and thus, we cannot confirm similar areas of improvement across the broader neuro-oncology AI literature. Nevertheless, findings in this article are similar to others [7, 20, 21] assessing areas of AI in oncology using CLAIM. However, the criteria may need to be weighted according to the importance of reporting items, and some manuscripts may not have been able to address every CLAIM criterion [11]. Lastly, assessing studies against the CLAIM criteria has an inherent degree of subjectivity, despite the steps we have taken to minimise this, such as the reviewing authors cross-checking each other’s assessments.

## Conclusion

The field of AI continues to evolve at a rapid pace. The availability of guidelines such as CLAIM allows for a more standardised approach to report quality for AI algorithms within the literature. From the articles reviewed in this study, high performance was observed across the four main AI tasks, but on average, assessed articles met less than half of the CLAIM criteria. The main areas of improvement include handling of data, ground truth, AI algorithm training, and validation. Introduction of the CLAIM criteria did not raise reporting standards as the adherence was still low between pre-CLAIM and post-CLAIM groups. Application of reporting standards such as the CLAIM will be an important means of addressing the translational gap between computer science research and clinical implementation.

## Supplementary Information

Below is the link to the electronic supplementary material.

Supplementary file1 (DOCX 35 KB)
