# Supplementary material for: Assessment of artificial intelligence (AI) reporting methodology in glioma MRI studies using the Checklist for AI in Medical Imaging (CLAIM)
Source: Neuroradiology. 2023 Feb 7;65(5):907–13. doi: 10.1007/s00234-023-03126-9 (PMC10105653; doi:10.1007/s00234-023-03126-9)
Supplement: Supplementary file 1 — Supplementary file1 (DOCX 35 KB) [file 234_2023_3126_MOESM1_ESM.docx]

**Appendix**

Table 1: CLAIM: Checklist for Artificial Intelligence in Medical Imaging. Courtesy of Mongan et. al. [11]

| Section / Topic | No. | Item |  |
| --- | --- | --- | --- |
| TITLE / ABSTRACT |  |  |  |
|  | **1** | Identification as a study of AI methodology, specifying the category of technology used (e.g., deep learning) |  |
|  | **2** | Structured summary of study design, methods, results, and conclusions |  |
| INTRODUCTION |  |  |  |
|  | **3** | Scientific and clinical background, including the intended use and clinical role of the AI approach |  |
|  | **4** | Study objectives and hypotheses |  |
| METHODS |  |  |  |
| *Study Design* | **5** | Prospective or retrospective study |  |
|  | **6** | Study goal, such as model creation, exploratory study, feasibility study, non-inferiority trial |  |
| *Data* | **7** | Data sources |  |
|  | **8** | Eligibility criteria: how, where, and when potentially eligible participants or studies were identified (e.g., symptoms, results from previous tests, inclusion in registry, patient-care setting, location, dates) |  |
|  | **9** | Data pre-processing steps |  |
|  | **10** | Selection of data subsets, if applicable |  |
|  | **11** | Definitions of data elements, with references to Common Data Elements |  |
|  | **12** | De-identification methods |  |
|  | **13** | How missing data were handled |  |
| *Ground Truth* | **14** | Definition of ground truth reference standard, in sufficient detail to allow replication |  |
|  | **15** | Rationale for choosing the reference standard (if alternatives exist) |  |
|  | **16** | Source of ground-truth annotations; qualifications and preparation of annotators |  |
|  | **17** | Annotation tools |  |
|  | **18** | Measurement of inter- and intrarater variability; methods to mitigate variability and/or resolve discrepancies |  |
| *Data Partitions* | **19** | Intended sample size and how it was determined |  |
|  | **20** | How data were assigned to partitions; specify proportions |  |
|  | **21** | Level at which partitions are disjoint (e.g., image, study, patient, institution) |  |
| *Model* | **22** | Detailed description of model, including inputs, outputs, all intermediate layers and connections |  |
|  | **23** | Software libraries, frameworks, and packages |  |
|  | **24** | Initialization of model parameters (e.g., randomization, transfer learning) |  |
| *Training* | **25** | Details of training approach, including data augmentation, hyperparameters, number of models trained |  |
|  | **26** | Method of selecting the final model |  |
|  | **27** | Ensembling techniques, if applicable |  |
| *Evaluation* | **28** | Metrics of model performance |  |
|  | **29** | Statistical measures of significance and uncertainty (e.g., confidence intervals) |  |
|  | **30** | Robustness or sensitivity analysis |  |
|  | **31** | Methods for explainability or interpretability (e.g., saliency maps), and how they were validated |  |
|  | **32** | Validation or testing on external data |  |
| RESULTS |  |  |  |
| *Data* | **33** | Flow of participants or cases, using a diagram to indicate inclusion and exclusion |  |
|  | **34** | Demographic and clinical characteristics of cases in each partition |  |
| *Model performance* | **35** | Performance metrics for optimal model(s) on all data partitions |  |
|  | **36** | Estimates of diagnostic accuracy and their precision (such as 95% confidence intervals) |  |
|  | **37** | Failure analysis of incorrectly classified cases |  |
| DISCUSSION |  |  |  |
|  | **38** | Study limitations, including potential bias, statistical uncertainty, and generalizability |  |
|  | **39** | Implications for practice, including the intended use and/or clinical role |  |
| OTHER INFORMATION |  |  |  |
|  | **40** | Registration number and name of registry |  |
|  | **41** | Where the full study protocol can be accessed |  |
|  | **42** | Sources of funding and other support; role of funders |  |
